# English Veterinary Colleges and Their Graduates

**Published:** 1883-01

**Authors:** 


English Veterinary Colleges and Their Graduates.—Dr. F S. Billings, in an able letter to the *Medical News*, gives us a picture of English veterinary schools, which is as true as it is unflattering. He says: "These English veterinary colleges are private institutions, utterly without responsibility, until the profession took its protection into its own hands by the formation of the Royal College of Veterinary Surgeons, which is acknowledged by the Government (only within a few years), and now constitutes the examining and diploma-granting body. Even this we can scarcely ever expect to arrive at in this country.

With reference to these British schools it should never be forgotten, that neither of them have ever produced any scientific contributions to veterinary literature, and that all that can be said of them is that they are and have been successful in producing good routine practitioners.

The English schools are, in reality, nothing more or less than speculative affairs, the benefits going to the teachers and the subscribers, who, for a certain sum per year can have the benefits of the school hospital. There is no very good feeling existing between the profession in the cities where these schools are, especially London, and the faculty, the schools frequently doing a 'cutting' business with regard to fees."

The above description tallies very well with what we have seen of many so-called English veterinarians. Individuals who attach M. R. C. V. S. to their names are getting to be quite numerous in this country. A few of them are excellent gentlemen who do credit to the profession, and these we heartily welcome. Many others are ignorant fellows, and it is a question sometimes whether they are really entitled to the degree they claim to possess. If so, the title of M. R. C. V. S. signifies very little, and the fact is becoming known. The graduates of our two veterinary colleges are, on the whole, much better educated men.

